# Multitasker Argonaute leaves no stone unturned

**DOI:** 10.1093/plcell/koae306

**Published:** 2024-11-16

**Authors:** Laura Arribas-Hernández

**Affiliations:** Assistant Features Editor, The Plant Cell, American Society of Plant Biologists; Consejo Superior de Investigaciones Científicas (CSIC), Instituto de Hortofruticultura Subtropical y Mediterránea ‘La Mayora’ (IHSM), Bulevar Louis Pasteur, 49, 29010 Málaga, Spain

Silencing genes in plants is not an easy task. First, the system must distinguish between the genes that deserve to be shut down from those that must remain active; such a distinction can be tricky. Second, the silencing machinery must get up and running to just the right extent: too little and plant development, antiviral defense, and stress responses would be in jeopardy; too much and silencing amplification mechanisms or immune responses can go into overdrive, even to the point of killing the plant. Hence, a highly specific and finely tuned silencing mechanism is essential for cells. Fortunately, plants have a whole family of dedicated Argonaute proteins, up to the task, at their disposal.

Argonautes are experts in silencing gene expression, but even experts have weaknesses. For Argonautes, one weak point may be that they rely on small RNAs (sRNAs) to guide them to their targets. If the source of sRNAs runs dry, the Argonaute would helplessly witness how undesirable targets become unleashed … or at least we used to believe so. Now, in new work in *The Plant Cell*, **Jing Li, Brandon Le, Xufeng Wang, and colleagues** (**Li et al. 2024**) report how Arabidopsis Argonaute1 (AGO1) can secure its own steady supply of some types of sRNAs by actively enhancing the transcription of Inverted Repeats (IRs) that constitute the source of such sRNAs. But how?

AGO1-mediated post-transcriptional gene silencing in the cytoplasm of plant cells is relatively well understood. Once a target transcript is bound to AGO1 through base-pairing to the AGO1-loaded sRNA, AGO1 can use either direct cleavage or translational repression to silence it. While the basic principles governing target cleavage were rapidly elucidated during the early 2000s, AGO1-mediated translational repression in plants, and indeed in any organism, has been a tougher nut to crack. It took years to identify the elusive plant AGO1 partner that would assist the silencing complex in repressing translation, until Shengben Li, with Xuemei Chen and colleagues, finally identified a crucial missing piece in 2013. They reported that the integral membrane protein ALTERED MERISTEM PROGRAM 1 (AMP1) associates with AGO1 at the endoplasmic reticulum to inhibit the translation of microRNA (miRNA, a type of sRNA) targets (Li et al. 2013). Importantly, these targets include *AGO1* mRNA itself, explaining why AGO1 protein is substantially more abundant in plants lacking AMP1 (Li et al. 2013) (Fig).

**Figure.**
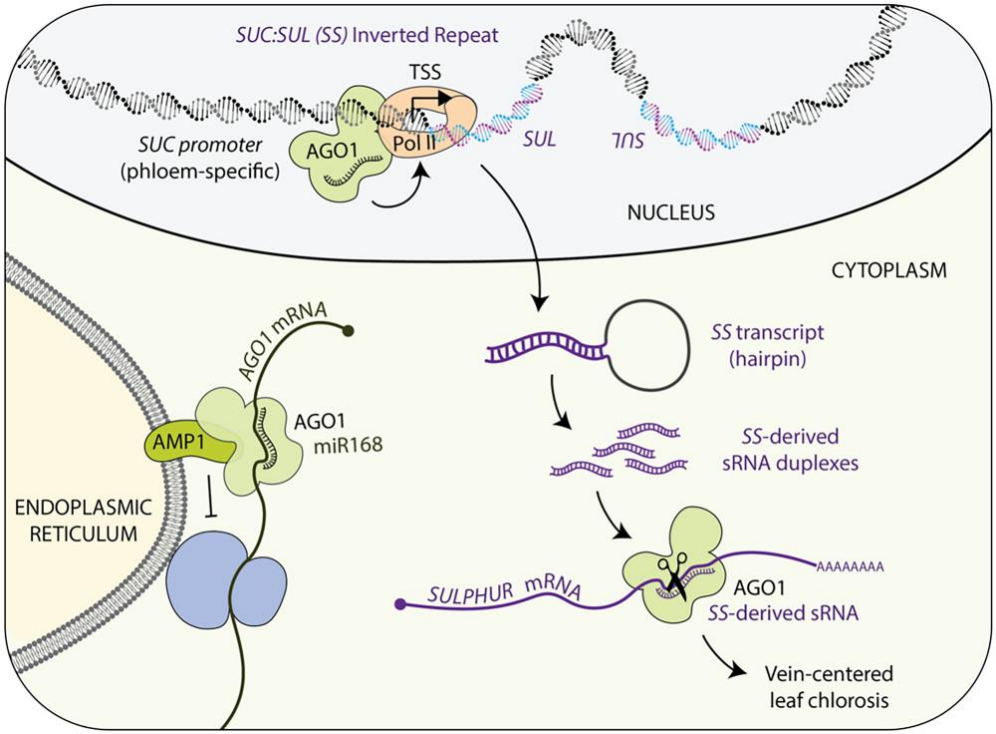
Major events in AGO1-catalyzed gene silencing. Homeostasis of AGO1 mRNA and protein involves autoregulation through AGO1 loaded with miR168, and the translational repression of AGO1 mRNA through AGO1-miR168 complexes involves AMP1 at the rough endoplasmic reticulum (lower left). Additionally, cytoplasmic AGO1 loaded with hairpin-derived sRNAs from IRs like *SUC:SUL* (*SS*) can silence complementary transcripts (*SULPHUR*) through canonical post-transcriptional gene silencing (PTGS) (lower right). A new piece in this puzzle is that nuclear AGO1 stimulates transcription of the source of sRNAs through association with chromatin at the transcription start sites (TSSs) of IR genes (top). In the absence of AMP1, abnormally high AGO1 levels caused by misregulation via miR168 cause an increase in AGO1 presence at TSSs of IRs, which in turn stimulates PTGS. Figure credit: L. Arribas-Hernández.

The nuclear activities of plant AGO1 are less well known. However, a model is emerging in which AGO1 can load certain sRNAs and even control their biogenesis in the nucleus (see Bajczyk et al. 2019 for a review). Interestingly, a study on chromatin-bound AGO1 reported an enrichment of AGO1 ChIP-Seq signal at transcription start sites of genes that are, on average, more highly expressed than non–AGO1-bound genes (Liu et al. 2018). Because they also found that RNA polymerase II (Pol II) occupancy and expression of these genes is generally reduced in *ago1-36* mutants, Liu et al. suggested that nuclear AGO1 promotes transcription of bound loci (Liu et al. 2018). Furthermore, based on the AGO1 ChIP-Seq profile in sRNA biogenesis mutants, the authors concluded that sRNAs are necessary for this action (Liu et al. 2018). However, it remained unclear whether a connection between such transcription-promoting activity in the nucleus and the well-established silencing function in the cytoplasm exists. Paradoxically, one would expect that the output of both actions would be, in principle, the opposite. Now, Li et al. (2024) have found that both activities may indeed be linked and can cooperate to achieve more efficient silencing.

While searching for a possible role of AMP1 and its paralog LIKE-AMP1 (LAMP1) in AGO1 functions beyond miRNA-mediated translational repression, Li et al. (2024) observed with surprise that IR-triggered silencing is exacerbated, rather than suppressed, in *amp1 lamp1* double mutants. In particular, the authors crossed *amp1 lamp1* plants to the widely used RNAi reporter line *SUC:SUL* (*SS*), in which a phloem-specific promoter (*SUC*) drives the expression of an artificial IR of the chlorophyl-biosynthesis gene *SULPHUR* (*SUL;* also known as *CHLORINA42*). *SS* transcripts give rise to sRNAs that target endogenous *SUL* (Fig), resulting in conspicuous vein chlorosis that is AGO1 dependent (Himber et al. 2003). The exacerbation of *SUL* silencing in *SS amp1 lamp1* plants manifests itself as a dramatic increase in both the chlorotic area on the leaves and the abundance of *SS*-derived sRNAs (Li et al. 2024). This overaccumulation is caused by a comparable increase in the abundance of the sRNAs precursor, the *SS* transcript, which in turn is produced by an elevated occupancy of initiating Pol II at the *SS* locus (Li et al. 2024). Interestingly, this increase also correlates with higher AGO1 occupancy at the *SS* locus in *amp1 lamp1* mutants, which is likely due to the overall high levels of AGO1 in these plants (Li et al. 2013). Indeed, the effect of a lack of AMP1/LAMP1 can be recapitulated by *AGO1* overexpression (Li et al. 2024). In other words, AGO1 acts not only as an effector of *SS*-derived sRNAs to silence the endogenous *SUL* gene, but also ensures a constant supply of *SS* sRNAs by promoting the transcription of their precursor (Fig).

Importantly, Li et al. demonstrated that the effect of *AMP1 LAMP1* mutation observed for the artificial *SS* system can be extended to endogenous inverted repeats, such as *IR71* or At5g22960 (Li et al. 2024). However, not all inverted loci behaved in this way, as other IR-derived siRNAs were unaffected or even showed reduced accumulation in *amp1 lamp1* mutant plants (Li et al. 2024).

The study by Li et al. (2024) is an important new piece in the puzzle of nuclear AGO1 functions, a puzzle that nevertheless remains incomplete. Among the missing pieces, the mechanism guiding AGO1 to the *SS* transgene or endogenous IR genes, and the molecular basis of AGO1-mediated stimulation of Pol II transcription at bound loci are particularly relevant problems that must await further studies.

## Data Availability

No new data were generated or analysed in support of this research.

## Dive Curated Terms

The following phenotypic, genotypic, and functional terms are of significance to the work described in this paper:

AGO1 Gramene: AT1G48410
AGO1 Araport: AT1G48410

MERISTEM AmiGo: PO:0009013

AMP1 Gramene: AT3G54720
AMP1 Araport: AT3G54720
